# Development and Validation of a Simple, Green Infrared Spectroscopic Method for Quantitation of Sildenafil Citrate in Siloflam Tablets of Unknown Manufacturing Formula

**DOI:** 10.1155/2021/6616728

**Published:** 2021-02-13

**Authors:** Van Trung Bui, Cao Son Doan, Thi Thanh Vuong Tong, Dinh Chi Le

**Affiliations:** ^1^National Institute of Drug Quality Control, Ministry of Health, Hanoi, Vietnam; ^2^Department of Analytical Chemistry and Toxicology, Hanoi University of Pharmacy, Hanoi, Vietnam

## Abstract

A simple, easy-to-implement, and green infrared spectroscopic method was developed and validated for the quantitative determination of sildenafil citrate in tablets of unknown manufacturing formula. Homogenized tablet powder with known mass content (%, m/m) of sildenafil citrate was mixed with paracetamol to form standard mixtures with different percentages of sildenafil citrate on the total quantity of sildenafil citrate and paracetamol (designated as R). Unknown tablet samples were finely ground and mixed with paracetamol to form test mixtures having *R* values about 50%. Infrared spectra of standard mixtures, measured in attenuated total reflectance mode, in the wavenumber zone from 1800 cm^−1^ to 1300 cm^−1^ were selected and processed by partial least square regression to form the calibration model for quantitation of sildenafil citrate in unknown samples. Spectral responses of test mixtures and the calibration model were used to determine the exact mass content (%, m/m) of sildenafil citrate in the powder of unknown tablet samples. The method was fully validated in terms of linearity, precision, and accuracy according to the requirements of current guidelines and was proved as reliable and suitable for the intended application.

## 1. Introduction

Sildenafil, usually used in form of citrate salt, is a phosphodiesterase-5 (PDE-5) inhibitor widely used for the treatment of erection disorder [1]. In Vietnam, products containing sildenafil are currently commercialized under many different brand names and mostly in tablet form, and the use of these products is largely depending on the personal need of users and not strictly controlled by obligated prescription [2]. In consequence, many products containing sildenafil available on market may come from dubious origins and are not legally distributed in pharmacies and their quality and safety can become a potential risk for users. This situation makes products containing sildenafil a prioritized target of postmarketing surveillance in Vietnam, including quality control. With the limited available resource of drug quality control network, the use of conventional techniques like HPLC, which is reliable but time- and resource-consuming, for routine control of a large number of samples taken from the market is not the optimal solution [3, 4]. A more feasible approach is to use some alternative technique acceptably reliable but faster and less resource-consuming, preferably also more friendly toward the environment, than HPLC for routine control of samples containing sildenafil taken from the market, reserving HPLC only for confirmative analysis of doubtful cases [5]. One of the most promising candidates for the role of alternative technique available nowadays is the group of analytical techniques deriving from infrared spectroscopy, including attenuated total reflectance infrared spectrometry (FTIR-ATR) [6, 7]. FTIR-ATR methods are fast and the spectra can be recorded directly on solid homogenized or liquid samples without the need for a time-consuming and complicated sample preparation process often necessary for conventional techniques (HPLC, UV-Vis spectrometry, etc.) [8, 9]. They are also economic in terms of operational cost per analysis and considered as green and friendly toward the environment because no consumption of organic solvents or chemicals is required [9]. So far, thanks to their advantages, FTIR-ATR has been employed for quantitative analysis of many types of analytes, including pharmaceutical drugs like anti-inflammatory drugs (etodolac, tolfenamic acid, bumadizone, and diacerein) [6] and ketoconazole [7]. In general, for quantitative analysis by FTIR-ATR, it is necessary to know the complete composition of the sample matrix to mix the matrix with analyte at different ratios to establish a calibration model [10, 11]. However, manufacturing formulas are confidential, so the state-own quality control laboratories have to deal frequently with commercialized samples of an unknown matrix. In this study, paracetamol was employed as an “internal standard” to establish the linear relationship between the mass ratio of sildenafil citrate over the total quantity of sildenafil citrate and paracetamol in the sample and the spectral responses, together with multivariable analysis to avoid intervention from unknown components.

## 2. Materials and Methods

### 2.1. Instrumentation

The infrared spectra were measured by a FT-IR spectrophotometer, Thermo Nicolet IS50 of Thermo Scientific (Waltham, MA, USA) in attenuated total reflectance mode. Software OMNIC version 9.8 was used for recording spectra and software TQ Analyst version 9.8 of Thermo Scientific (Waltham, MA, USA) was used to process spectra and establish a calibration model.

An Agilent 1200 HPLC system of Agilent Technologies (Santa Clara, CA, USA) equipped with a PDA detector was used for determining the exact content (%, m/m) of sildenafil citrate in stock standard material (see 2.3.2. for detailed description). The chromatographic separation was executed on a Luna C18 column (250 × 4.6 mm, 5 *µ*m) of Phenomenex (CA, USA). Software ChemStation Version B.04.03 was used for recording and processing chromatograms. Analytical balance MS105 of Mettler Toledo (Columbus, OH, USA) with readability 0.01 mg was used to determine average tablet weight and weighing powder to form mixtures.

### 2.2. Chemicals and Reagents

Reference substances of sildenafil citrate (purity 98.9%) were established internally at the National Institute of Drug Quality Control (Hanoi, Vietnam). Pharmaceutical grade paracetamol (purity 99.7%) was purchased from Farmson Pharmaceutical Pvt. Ltd. (Gujarat, India). HPLC grade acetonitrile and PA grade potassium dihydrogenphosphate were purchased from Merck Vietnam (Ho Chi Minh City, Vietnam). Siloflam tablets (manufactured by Flamingo Pharmaceuticals Ltd., Mumbai, India, containing nominally 100 mg of sildenafil (equivalent to 140.5 mg of sildenafil citrate) per tablet belonging to batches N531 and N532 were purchased from the market. Siloflam tablets were selected for this study because we did not have any information regarding their exact excipient composition and manufacturing formula; therefore, this product was ideal for assessing the feasibility and reliability of using paracetamol as an internal standard for FT-IR assay of sildenafil in tablets of an unknown matrix.

### 2.3. Standard and Test Preparations

#### 2.3.1. For HPLC Analysis

*(1) Standard Solution*. A solution containing an accurate concentration of sildenafil citrate about 50 *µ*g/ml in the mobile phase was used as the standard solution.

*(2) Test Solution*. Twenty Siloflam tablets randomly selected were finely ground and homogenized. An accurately weighed quantity of tablet powder was dissolved in the mobile phase to obtain a sample solution having a concentration of sildenafil citrate about 50 *µ*g/ml in the mobile phase.

#### 2.3.2. For IR Analysis

*(1) Stock Standard Material*. Twenty Siloflam tablets from the batch N532 were accurately weighed to determine the average weight per tablet and then finely ground to form a homogenized powder (stock standard material). The mass content of sildenafil citrate (%, m/m) in stock standard material was determined by using a validated HPLC method (see details in 3.1) and was found to be 37.94% (m/m).

*(2) Working Standard Mixtures*. Different portions of stock standard material containing 37.94% (m/m) of sildenafil citrate were homogenously mixed with paracetamol to form working standard mixtures. For each standard mixture, the percentage of sildenafil citrate on the total quantity of sildenafil citrate and paracetamol was designated as *R* (%). Working standard mixtures were prepared in duplicate at 5 different levels of *R* values (about 30%, 40%, 50%, 60%, and 70%).

*(3) Test Mixtures*. Siloflam tablets with an unknown mass content of sildenafil citrate were finely ground and homogenized. The estimated mass content of sildenafil citrate (%, m/m) in the sample was calculated from the labeled amount of sildenafil citrate per tablet and the average weight per tablet. The sample powder was then mixed with paracetamol to obtain test mixtures with an estimated *R* value of about 50%.

For method validation, stock standard material was mixed with paracetamol to form mixtures with *R* values of about 35%, 50%, and 65%.

### 2.4. Method

#### 2.4.1. HPLC Analysis

The quantification of sildenafil citrate in stock standard material (see 2.3.2) by HPLC was done with a Luna C18 column using a mixture of acetonitrile and 0.05 M aqueous solution of potassium dihydrogenphosphate (70: 30, v/v) as mobile phase. The flow rate of the mobile phase was maintained at 1.0 ml per minute. The detection was done at wavelength 230 nm. The injection volume was 20 *µ*l.

#### 2.4.2. IR Analysis

The infrared spectra of working standard mixtures and test mixtures (see 2.3.2) were measured in ATR mode in the wavenumber zone from 2000 cm^−1^ to 400 cm^−1^, with wavenumber resolution 4 cm^−1^. To record spectra for establishing calibration model, each working standard mixture was scanned 10 times independently at different positions. For analysis of unknown samples, each test mixture was scanned 5 times independently at different positions.

#### 2.4.3. Chemometric Analysis of Infrared Spectra

After measurements, raw spectra were normalized at wavenumber 1697.6 cm^−1^ before processing to reduce the effect of unstable intensity in ATR mode. In TQ Analyst software, spectra were processed in Quantitative Analysis Type using partial least square (PLS) regression. Pathlength Type is multiplicative signal correction (MSC). Spectra of working standard mixtures were added on software together with their respective *R* values and used in the original data format (spectrum option). In order to reduce the effect of tablet matrix, the spectra were investigated by the software to find out the wavenumber zone where the signal from the tablet matrix is low and the main spectral signals come from sildenafil citrate and paracetamol. After investigation, the wavenumber zone from 1800 cm^−1^ to 1300 cm^−1^ was selected for quantitative analysis of sildenafil citrate in tablets. To control the overfitting phenomenon, spectra obtained from working standard mixtures were divided randomly into calibration set and validation set. A calibration model would be established based on the balance of correlation coefficient factor and root mean square error (RMSE) between calibration set and validation set.

To determine mass content (%, m/m) of sildenafil citrate in tablet powder sample, IR spectra of each test mixture were randomly recorded at 5 different positions. After normalization at 1697.6 cm^−1^, these spectra were applied to the calibration model to determine the *R* values.

Mass content of sildenafil citrate in unknown Siloflam tablet powder (%, m/m) was calculated by using the following formulation:

(1) $$\begin{array}{c} C=\frac{R\times m_{\text{par}}\times P_{\text{par}}}{\left(100-R\right)\times m_{p}}. \end{array}$$

C was the mass content of sildenafil citrate in unknown Siloflam tablet powder, *R* was the found *R* value of test mixture, *m*_par_, *m*_*p*_ were the weight (in mg) of paracetamol and Siloflam tablet powder, respectively, in the test mixture, and *P*_par_ was the purity of paracetamol.

The reliability of the quantitative results obtained from the method was validated according to the current requirements of ICH guideline Q2R1 [12] and AOAC International [13] in terms of specificity, linearity, precision, and accuracy [14–18].

### 2.5. Data Processing

TQ Analyst software (version 9.8) of Thermo Scientific (Waltham, MA, USA) was used to process spectra and establish a calibration model. SPSS software (version 16.0) of IBM SPSS Software (IBM, Armonk, NY, USA) was used for statistical analysis of analytical results.

## 3. Results and Discussion

### 3.1. Determination of Sildenafil Citrate Content in Stock Standard Material

Twenty Siloflam tablets randomly selected from the N532 batch (average weight 360.1 milligrams per tablet after removing tablet coating) were finely ground and mixed to homogenize to form the stock standard material (see 2.3.2). The exact mass content of sildenafil citrate in stock standard material was quantified against reference standard by using the HPLC method described in 2.4.1. Quantitation results by HPLC revealed that stock standard material contained 37.64% (m/m) sildenafil citrate. This value was employed to calculate the *R* values of working standard mixtures.

### 3.2. Development of Calibration Model from Infrared Spectra

As an approach to overcome possible interference from the unknown composition in the manufacturing formula of Siloflam tablets, exact quantities of paracetamol were added and homogenously mixed with tablet powder at different mass ratios to play the role of the internal standard. Thanks to the presence of paracetamol, it was possible to establish a direct relationship between the variation in the mass ratio of sildenafil citrate and paracetamol in the sample and the variation of IR spectral response without knowing the exact composition of the tablet.

**Figure 1:**
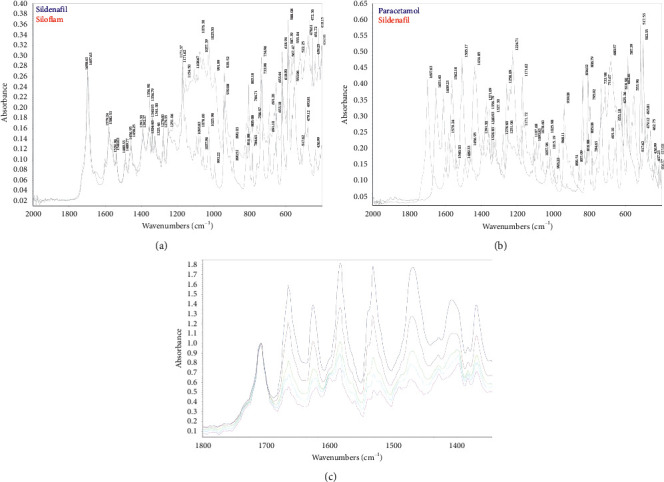
Comparing IR spectra of sildenafil citrate and Siloflam tablet powder (a). IR spectra of sildenafil citrate and paracetamol (b). IR spectra of some working standard mixtures with different *R* values (c).

Working standard mixtures were prepared with *R* values described in Table 1. Each mixture was scanned 10 times at 10 different positions to record IR spectra. From spectra of Siloflam tablet powder and sildenafil citrate powder, the wavenumber zone from 1800 cm^−1^ to 1300 cm^−1^ showed a low effect of sample matrix on the spectral signal of sildenafil citrate (Figure 1(a)). Furthermore, several wavenumbers corresponding to high absorbance in of paracetamol spectrum such as 1651.4 cm^−1^, 1609.2 cm^−1^, 1562.2 cm^−1^, 1505.2 cm^−1^, and 1434.9 cm^−1^ were also located in this range (Figure 1(b)). These maximal wavenumbers of paracetamol have also differed from those of sildenafil citrate located in the same wavenumber zone, including the wavenumber corresponding to the most intense maximum of sildenafil citrate at about 1697.6 cm^−1^. Thanks to these above-mentioned characteristics, within the wavenumber zone from 1800 cm^−1^ to 1300 cm^−1^, the main absorbance peaks of sildenafil citrate and paracetamol are not overlapped together; therefore, the variation of the mass ratio of sildenafil citrate on the total amount of sildenafil citrate and paracetamol in a mixture will likely cause a proportional variation in infrared spectrum within this zone. As a consequence, the selected wavenumber zone was representative for paracetamol and sildenafil mass ratio in the sample and applying spectral data processing on this zone would reduce the effect of the unknown sample matrix.

**Figure 2:**
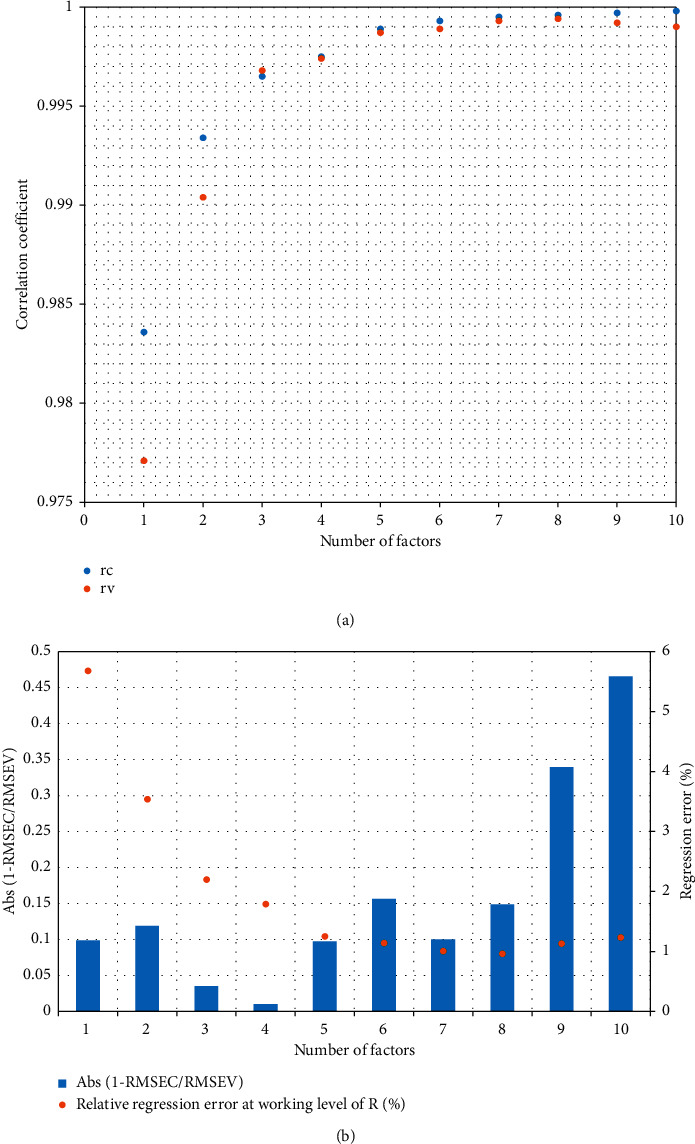
Selection of factor number for calibration model: (a) variation of correlation coefficient and (b) variation of the difference between RMSEC and RMSEV and variation of relative error due to linear regression at R value of about 50%).

**Figure 3:**
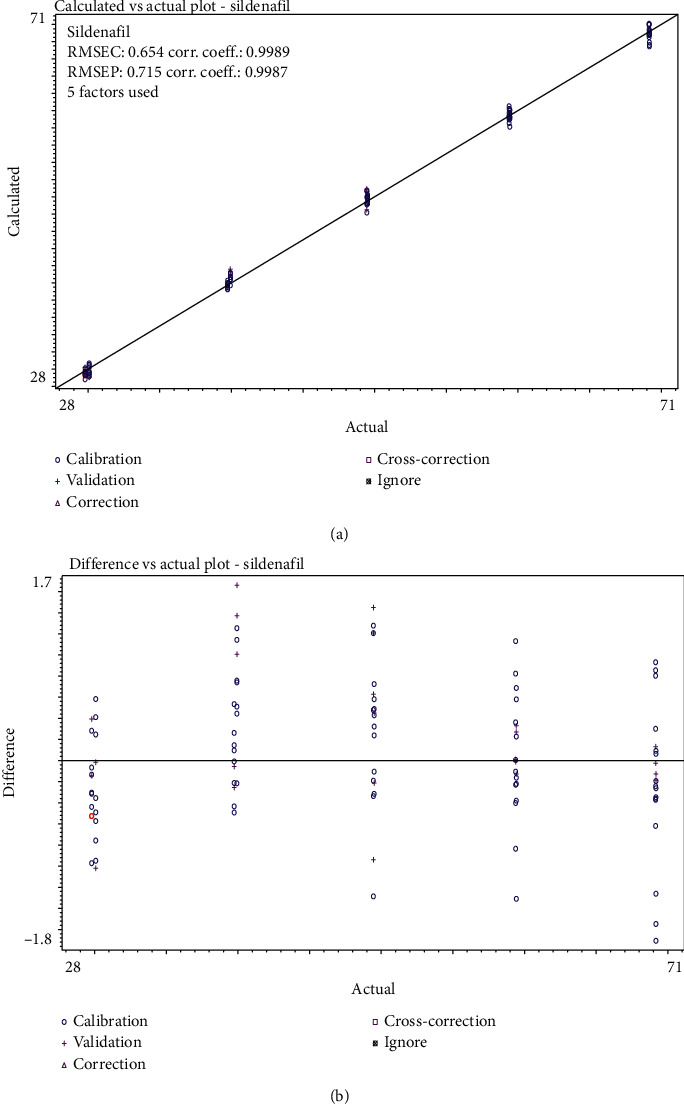
Calibration model: (a) regression curve and (b) residual chart.

To establish a calibration model, the spectra obtained from the working standard were divided randomly into calibration set (8 spectra) and validation set (2 spectra) at each *R* value level. The calibration set spectra were used to establish the regression relationship, and the validation set spectra were used for cross-validation of the model. Calibration models using partial least square regression with different numbers of factors were tried; the *R* values obtained from each model were compared to respective true *R* values of each working standard mixture belonging to calibration set and validation set to calculate the root mean square error of calibration (RMSEC) and that of validation (RMSEV), the correlation coefficients on calibration set (*r*_c_) and on the validation set (*r*_v_), and the relative error caused by linear regression process at *R* level of test mixture (about 50%). The modeling trials revealed that it was necessary to use at least 5 factors to obtain a correlation coefficient equal to or higher than 0.998 (see Figure 2(a)). But when the number of used factors went beyond 5 factors, the difference between RMSEC and RMSEV increased, signaling a decrease in prediction power for *R* value of unknown samples (see Figure 2(b)). The relative error due to linear regression at the working level of *R* (approximately 50%) was reduced to approximately 1% when 5 factors or more were employed in the calibration model, but employing more than 5 factors did not reduce it further significantly (see Figure 2(b)). Therefore, a calibration model employing 5 factors was selected for the final method (the regression curve and residual chart of this model were presented in Figures 3(a) and 3(b), resp.).

### 3.3. Method Validation

#### 3.3.1. Specificity

As being mentioned above, within the wavenumber zone from 1800 cm^−1^ to 1300 cm^−1^, the infrared spectra of mixtures containing Siloflam tablet powder and paracetamol depended mostly on the infrared absorbance of sildenafil citrate and paracetamol (see also Figures 1(a) and 1(b)). Thanks to the specificity of infrared spectra of sildenafil citrate and paracetamol, the spectral data profiles used to develop calibration model can appear only with the simultaneous presence of sildenafil citrate and paracetamol in the sample, and the calibration model was established between spectral response and the mass ratio of sildenafil citrate over total quantity of sildenafil citrate and paracetamol in the sample. Therefore, this method was specific for the assay of sildenafil citrate in Siloflam tablets [12, 13].

#### 3.3.2. Linearity and Range

According to guideline Q2R1 of ICH [12, 13], a quantitative method must maintain its linearity at least within the range from 80% to 120% of working concentration. In this study, the working value of *R* in test mixture was selected at around 50%.

The calibration model established from working standard mixtures showed that the linearity between *R* values and the spectral response was maintained within the range of *R* from 30% to 70%, that is, from 60% to 140% of the working value of *R* (see Figure 2(a)). The correlation coefficient of the calibration model was 0.999, higher than 0.998 for both calibration set and validation set of working standard mixtures (as presented in Figure 2(a)).

#### 3.3.3. Accuracy

The accuracy of an analytical method expresses the closeness of results obtained by that method to the true value. In this study, the results of recovery studies gave recovery rate from 98.1% to 101.6% at all three levels of *R* values, and RSD values at each level of *R* value varied from 0.9 to 1.3%, as shown in Table 2. These results were within the accepted limit for recovery (98.0% to 102.0%) and RSD (not more than 2.0%) [12, 13].

#### 3.3.4. Precision

The repeatability and the intermediate precision of the method were evaluated through the dispersion of quantitative results of the same sample obtained from independent analyses on the same day and on different days, respectively. The RSD of quantitative results (*n* = 6) in a single day was 1.6% and in two different days was 1.6% (*n* = 12), as presented in Table 3 which were satisfied in terms of precision for a quantitative method [12, 13].

### 3.4. Application

**Figure 4:**
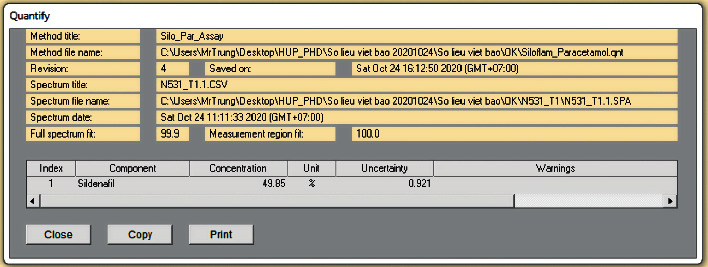
Determining R value of mixture of N531 powder and paracetamol.

To further assessment of the suitability of the method, the calibration model established with working standard mixtures described in 3.1 was used to quantify sildenafil citrate in Siloflam tablets batch N531 (a typical response window was presented in Figure 4). The average mass content sildenafil in the tablet was 38.1% (m/m) (*n* = 6, RSD = 1.4%, detailed results in Table 4), equivalent to 100.6% of average mass content of sildenafil citrate of the same batch obtained with HPLC method (37.6%) (assay results obtained with HPLC and typical chromatograms were provided in a separate Supplementary File and not included in this paper). The relative difference in assay results between IR method and HPLC method was 0.6%, within the accuracy limit from 98.0 to 102.0% imposed by AOAC International [13], whereas the acceptable variation in the content of sildenafil citrate in the tablet was 90.0 to 110.0% of the labeled amount [3, 13]. Therefore, it is possible to consider this IR method as an alternative technical choice besides the HPLC method for the quantitation of sildenafil citrate in Siloflam tablets.

### 3.5. Discussion

Up to now, HPLC-based methods have been and still are the main workhorse in drug quality control [5]. However, these methods are time-consuming, require a suitable sample preparation process which can be quite complicated for solid samples or analytes with poor solubility in common solvents, and produce an important quantity of organic solvent waste, which is highly polluted for the environment. A recent development in the methodology of spectroscopic analytical methods, including infrared spectroscopic ones, rendered these methods more suitable for routine application like quality control of commercialized drug in postmarketing surveillance. In this study, the use of paracetamol as an internal standard in solid state helped overcome the possible interference caused by the unknown excipient matrix of the Siloflam tablet and rendered possible the quantitative analysis of sildenafil citrate in Siloflam tablets directly in powder, without the need of any dissolution or extraction step. Application results obtained with another batch of Siloflam tablets (as described in 3.3) also confirmed the reliability of this approach for a single unknown tablet matrix. For quality control of the pharmaceutical product, the assay of active principles (like sildenafil citrate) must be done on a homogenized sample to assure the representative of results. Therefore, with coated tablets like Siloflam ones, the removal of the coating layer and homogenizing by fine grinding and mixing of an acceptable number of tablets (normally 20 ones) are obligated in the sample preparation process, regardless of the analytical techniques. So, in case of the FT-IR method employing solid internal standard, the most time-consuming step is the establishment of the calibration model. Once the model is established, it can be used for routine analysis of all other batches of Siloflam tablets. In the routine analysis, it is necessary only to mix homogenized powder of Siloflam tablet with IS (paracetamol) before measuring FT-IR spectra. In ATR mode, the record of FT-IR spectra is simple and rapid, so as a whole, routine analysis by FT-IR is still less labor- and time-consuming in terms of sample preparation and measurement than common techniques like UV-Vis spectroscopy or HPLC. With acceptable reliability in terms of linearity, precision, and accuracy, not requiring the use of an organic solvent or any other polluted chemical, the infrared spectroscopic method developed in this paper was a “green” solution for quantitative analysis of sildenafil citrate in tablets suitable for routine application in drug quality control, at least on a product-by-product basis.

However, in order to establish a more universal calibration model for quantitative analysis of sildenafil citrate by FT-IR in tablets, it will be necessary to employ more extensive standard stock materials coming from different brand names of tablets containing sildenafil citrate with different manufacturing formulas to find out the most suitable spectral zone, chemometric treatment of data, and regression model.

## 4. Conclusion

With the use of paracetamol as an internal standard and application of PLS regression for spectral data processing, a rapid, “green” IR spectroscopic method has been developed for the assay of sildenafil citrate in Siloflam tablets. The method was validated according to ICH guidelines and proved to be suitable for the intended application and able to provide accurate and precise quantitative results without the need of employing hazardous organic solvents and other chemicals unfriendly to the environment [18].

## Figures and Tables

**Table 1:** Working standard mixtures for calibration model.

Number	Quantity of Siloflam tablet powder (containing 37.94% (m/m) sildenafil citrate) (mg)	Quantity of paracetamol (purity: 99.7%) (mg)	*R* value (%)
1	156.93	140.97	29.79
2	159.41	141.28	30.08
3	209.88	120.36	39.93
4	206.32	119.26	39.74
5	260.25	101.43	49.45
6	257.91	100.28	49.51
7	309.85	80.65	59.43
8	308.75	80.53	59.38
9	361.28	61.46	69.15
10	360.36	61.23	69.17

**Table 2:** Results of accuracy.

Recovery study level	Replicate	Quantity Siloflam tablet powder (containing 37.94% sildenafil citrate) (mg)	Quantity of paracetamol (purity 99.7%) (mg)	Found *R* value of mixture (%)	Sildenafil citrate recovery (mg)	Recovery rate (%)	Mean recovery, RSD (%)
1 (*R* value about 35%)	1	180.46	131.07	34.41	68.54	100.1	100.1
	2	180.35	131.52	34.06	67.74	99.0	
	3	180.53	131.64	33.86	67.18	98.1	
	4	184.16	129.62	35.44	70.95	101.6	
	5	181.18	130.13	34.93	69.63	101.3	
	6	182.42	130.21	34.90	69.58	100.5	1.3

2 (*R* value about 50%)	1	268.15	99.28	51.02	103.10	101.3	100.6
	2	268.38	99.25	50.97	102.86	101.0	
	3	266.25	99.06	50.93	102.51	101.5	
	4	268.92	99.33	51.02	103.16	101.1	
	5	263.55	100.29	49.72	98.89	98.9	
	6	267.32	99.74	50.38	100.96	99.5	1.1

3 (*R* value about 65%)	1	335.69	71.68	64.15	127.88	100.4	99.8
	2	334.62	71.22	63.77	124.96	98.4	
	3	339.25	69.43	65.45	131.14	101.9	
	4	337.47	71.51	64.00	126.77	99.0	
	5	334.18	71.56	63.87	126.10	99.5	
	6	335.22	71.89	63.86	126.64	99.6	1.2

**Table 3:** Results of repeatability and intermediate precision.

Number of sample solutions	Sample weight (mg)	Paracetamol weight (mg, purity: 99.7%)	Calculated *R* value (%)	Mass content of sildenafil in tablet (%, m/m)
*Day 1, analyst 1*				
1	268.15	99.28	51.02	38.4
2	268.38	99.25	50.97	38.3
3	266.25	99.06	50.93	38.5
4	268.92	99.33	51.02	38.4
5	263.55	100.29	49.72	37.5
6	267.32	99.74	50.38	37.8
Average (1–6)				38.2
RSD (%) (1–6)				1.1

*Day 2, analyst 2*				
7	268.12	100.56	50.26	37.8
8	266.32	100.32	50.30	38.0
9	269.15	100.38	50.36	37.7
10	268.06	100.66	50.69	38.5
11	264.35	101.43	49.37	37.3
12	265.43	100.27	50.90	39.0
Average (1–12)				38.1
RSD (%) (1–12)				1.6

Results obtained on day 1 by analyst 1 (sample numbers 1–6) were used for evaluating repeatability and those obtained on day 1 and day 2 (sample numbers 1–12) were used together for evaluating intermediate precision.

**Table 4:** Results of quantitation on batch N531.

Replicates	Sample weight (mg)	Paracetamol weight (mg, purity: 99.7%)	Calculated *R* value (%)	Mass content of sildenafil in tablet (%, m/m)
1	267.28	100.08	49.95	37.3
2	269.33	100.21	50.88	38.4
3	270.14	100.13	50.53	37.7
4	269.66	100.22	50.86	38.3
5	268.47	100.37	50.36	37.8
6	268.35	100.04	51.06	38.8
Average (1–6)				38.1
RSD (%) (1–6)				1.4

## Data Availability

The data used to support the findings of this study are available from the corresponding author (ledinhchi@gmail.com) upon request. A separate Supplementary File containing assay results obtained with HPLC and typical chromatograms was also provided.

## Supplementary Materials

Supplementary Materials A separate supplementary file containing assay results obtained with HPLC and typical chromatograms is also provided. Table S1: results of quantitation. Figure S1. chromatogram of the standard solution (a), sample solution of batch N531 (b), and sample solution batch N532 (c).
