# Supplementary material for: Development and Validation of a Simple, Green Infrared Spectroscopic Method for Quantitation of Sildenafil Citrate in Siloflam Tablets of Unknown Manufacturing Formula
Source: J Anal Methods Chem. 2021 Feb 13;2021:6616728. doi: 10.1155/2021/6616728 (PMC7896866; doi:10.1155/2021/6616728)
Supplement: Supplementary Materials — A separate supplementary file containing assay results obtained with HPLC and typical chromatograms is also provided. Table S1: results of quantitation. Figure S1. chromatogram of the standard solution (a), sample solution of batch N531 (b), and sample solution batch N532 (c). [file 6616728.f1.docx]

Development and validation of a simple, green infrared spectroscopic method for quantitation of sildenafil citrate in Siloflam tablets of unknown manufacturing formula

Van Trung Bui^1^, Cao Son Doan^1^, Thi Thanh Vuong Tong^2^, Dinh Chi Le^2^*

^1^National Institute of Drug Quality Control, Ministry of Health, Vietnam

^2^Department of Analytical Chemistry and Toxicology, Hanoi University of Pharmacy, Vietnam

*Corresponding author, email: [ledinhchi@gmail.com](mailto:ledinhchi@gmail.com)

Abstract

A simple, easy-to-implement and green infrared spectroscopic method was developed and validated for quantitative determination of sildenafil citrate in tablets of unknown manufacturing formula. Homogenized tablet powder with known mass content (%, m/m) of sildenafil citrate was mixed with paracetamol to form standard mixtures with different percentages of sildenafil citrate on the total quantity of sildenafil citrate and paracetamol (designated as R). Unknown tablet samples were finely ground and mixed with paracetamol to form test mixtures having R values about 50%. Infrared spectra of standard mixtures, measured in attenuated total reflectance mode, in the wavenumber zone from 1800 cm^-1^ to 1300 cm^-1^ were selected and processed by partial least square regression to form the calibration model for quantitation of sildenafil citrate in unknown samples. Spectral responses of test mixtures and the calibration model were used to determine the exact mass content (%, m/m) of sildenafil citrate in the powder of unknown tablet samples. The method was fully validated in terms of linearity, precision, accuracy according to requirements of current guidelines and was proved as reliable and suitable for the intended application.

Keyword: sildenafil, ATR-FTIR spectrometry, partial least square, multiplicative signal correction.

**HPLC RESULTS FOR QUANTITATIVE SILDENAFIL CITRATE**

**IN SILOFLAM TABLET**

1. Materials and methods

An Agilent 1200 HPLC system of Agilent Technologies (Santa Clara, CA, USA) equipped with a PDA detector was used for determination the exact content (%, m/m) of sildenafil citrate in stock standard material. The chromatographic separation was executed on a Luna C18 column (250 × 4.6 mm, 5 µm) of Phenomenex (CA, USA). Mobile phase was a mixture of acetonitrile and 0.05 M aqueous solution of potassium dihydrogenphosphate (70 : 30, v/v) maintained at flow rate of 1.0 mililitre per minute. Injection volume was 20 μL. UV detection was realized at 230 nm.

Software ChemStation Version B.04.03 was used for recording and processing chromatograms. Analytical balance MS105 of Mettler Toledo (Columbus, OH, USA) with readability 0.01 mg was used to determine average tablet weight and weighing powder to form mixtures.

2. Chemicals and reagents

Reference substances of sildenafil citrate (purity 98.9%) was established internally at National Institute of Drug Quality Control (Hanoi, Vietnam). Pharmaceutical grade paracetamol (purity 99.7%) was purchased from Farmson Pharmaceutical Pvt. Ltd. (Gujarat, India). HPLC grade acetonitrile and PA grade potassium dihydrogenphosphate were purchased from Merck Vietnam (Ho Chi Minh City, Vietnam). Siloflam tablets (manufactured by Flamingo Pharmaceuticals Ltd., Mumbai, India, containing nominally 100 mg of sildenafil (equivalent to 140.5 mg of sildenafil citrate) per tablet belonging to batches N531 and N532 were purchased from market.

3. Results

The average of peak area of standard solution (prepared by dissolved 50.02 mg sildenafil citrate in 100.0 ml mobile phase) was 2718.3;

Mass content of sildenafil citrate in the two study batch were presented in Table S1.

Table S1. Results of quantitation

| Replicates | Sample weight (mg) | Pic area  (mAU) | Mass content of sildenafil citrate in tablet (%, m/m) |
| --- | --- | --- | --- |
| Batch: N531 (weight average = 360.08 mg after removing tablet coating) | | | |
| 1 | 132.48 | 2750.8 | 37.79 |
| 2 | 133.12 | 2731.6 | 37.34 |
| 3 | 133.35 | 2762.1 | 37.70 |
| **Average (1-3)** |  |  | **37.61** |
| RSD (%)(1-3) |  |  | 0.6 |
| Batch: N532 (weight average = 360.12 mg after removing tablet coating) | | | |
| 1 | 127.27 | 2654.9 | 37.96 |
| 2 | 130.51 | 2709.1 | 37.78 |
| 3 | 132.45 | 2770.9 | 38.07 |
| **Average (1-3)** |  |  | **37.94** |
| RSD (%)(1-3) |  |  | 0.4 |


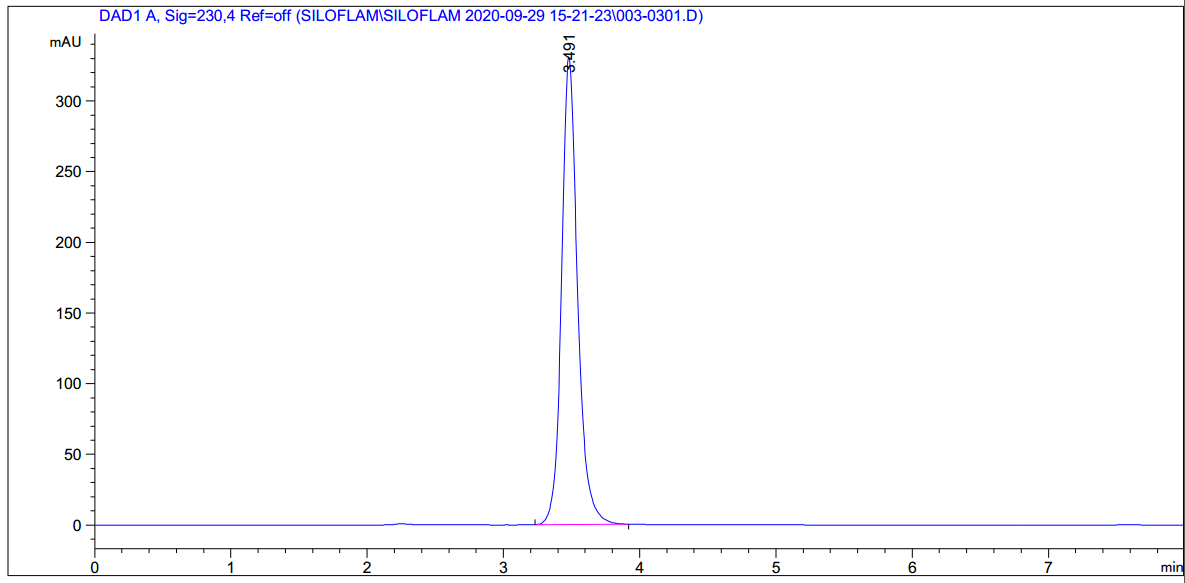


**a)**


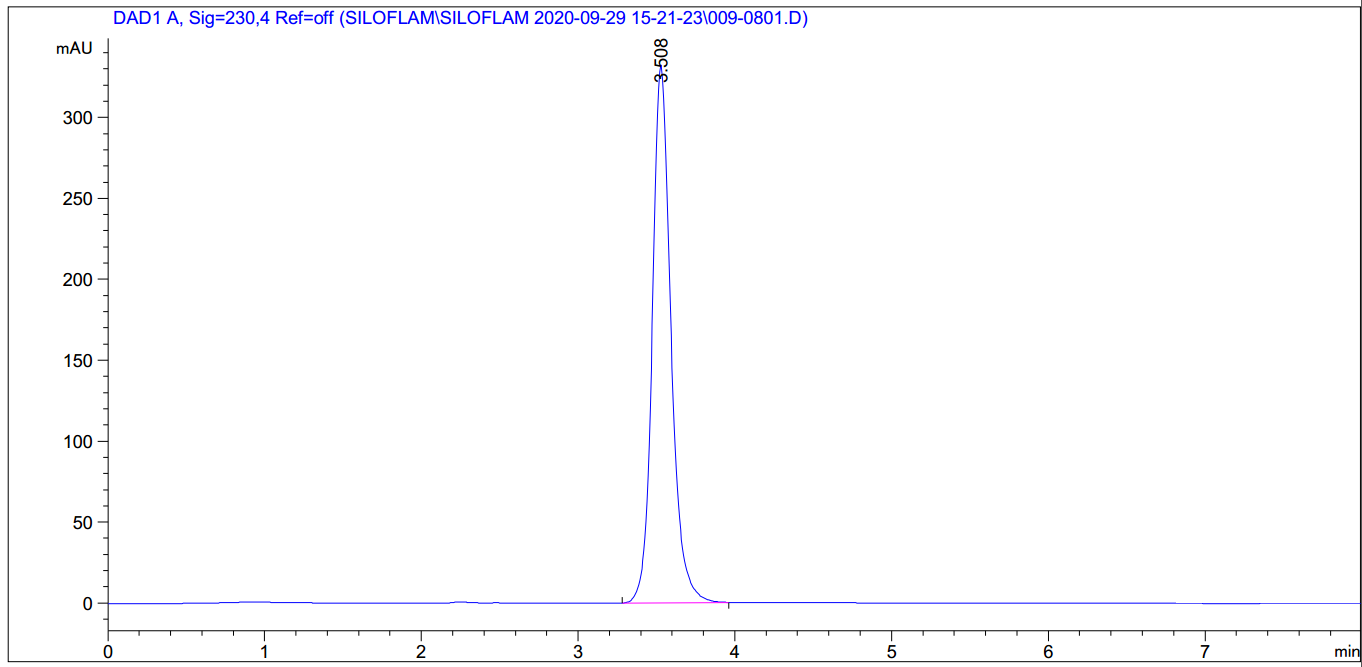


**b)**


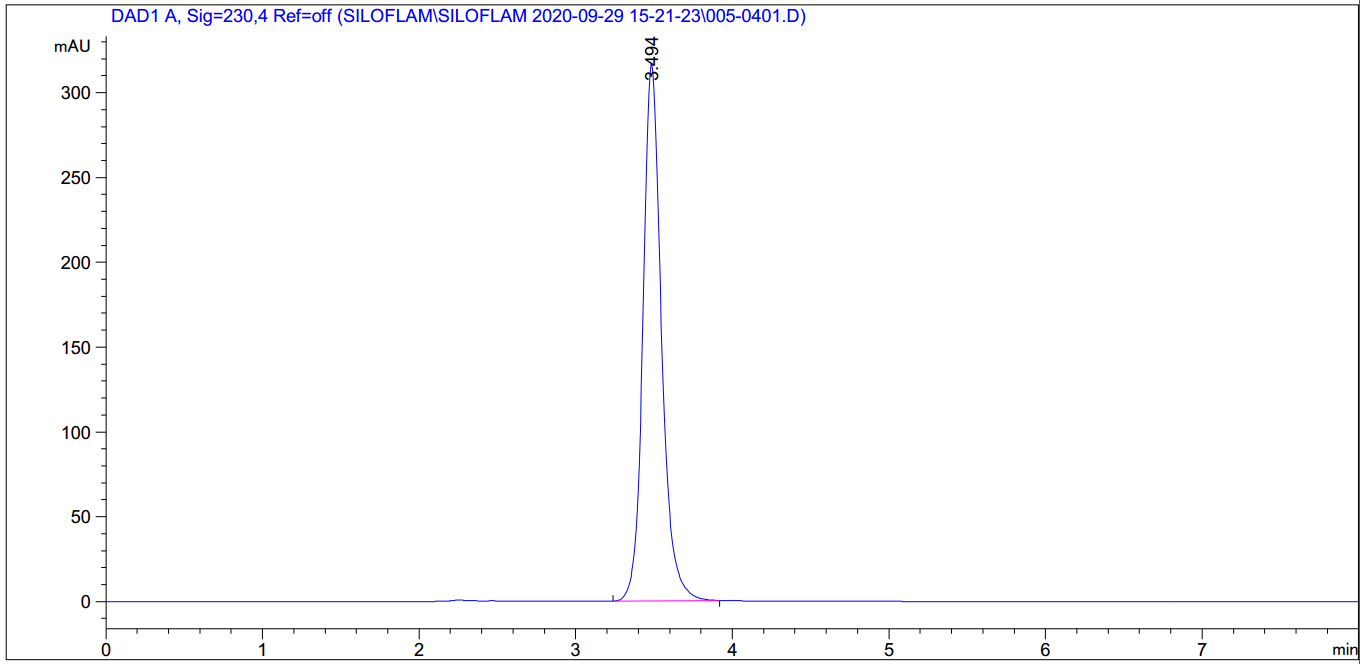


**c)**

Figure S1. Chromatogram of Standard solution (a), Sample solution of batch N531 (b) and Sample solution batch N532 (c).
